# Data on Heavy metal in coastal sediments from South East Coast of Tamilnadu, India using Energy Dispersive X-ray Fluorescence (EDXRF) Technique

**DOI:** 10.1016/j.dib.2016.09.047

**Published:** 2016-10-06

**Authors:** J. Chandramohan, G. Senthilkumar, M. Suresh Gandhi, R. Ravisankar

**Affiliations:** a Department of Physics, CK College of Engineering & Technology, Cuddalore 607003, Tamilnadu, India; b Department of Physics, University of College of Engineering Arni (A Constituent College of Anna University), Arni 632326, Tamil Nadu, India; c Department of Geology, University of Madras Guindy Campus, Chennai 600025, Tamilnadu, India; d PG & Research Department of Physics, Government Arts College, Thiruvannamalai 606603, Tamilnadu, India

**Keywords:** Sediment, Heavy metals, EDXRF, Tamilnadu Coast

## Abstract

This article contains the chemical and geographical data and figures for the chemical data in sediments of East Coast (Pattipulam to Dhevanampattinam) of Tamilnadu. The obtained data are related to the research article “Heavy Metal Assessment in Sediment Samples Collected From Pattipulam to Dhevanampattinam along the East Coast of Tamil Nadu Using EDXRF Technique” (Chandramohan et al., 2016) [1]. Chemical data are collected from Energy dispersive X-ray fluorescence spectrometer (EDXRF). Furthermore, the obtained chemical data describes it in more detail in the figures.

**Table:** 

Subject area	Physics, Chemistry, Geology
More specific subject area	Heavy metal contents in marine environment
Type of data	Table, Figures
How data was acquired	Energy Dispersive X-ray Fluorescence Spectrometer (EDXRF)
Data format	Raw
Experimental factors	The sediment samples were dried at 105 °C for 24 h, homogenized and sieved using a 63 μm in order to identify the geochemical concentrations. All powder samples were stored in desiccators until they were analyzed.One gram of the fine grinded sample and 0.5 g of boric acid (H_3_BO_3_) was mixed. The mixture was thoroughly grinded and pressed to a pellet of 25 mm diameter using a hydraulic press (20 tons) for EDXRF analysis.
Experimental features	Determination of concentration of Mg, Al, K, Ca, Ti, Fe, V, Cr, Mn, Co, Ni, Zn, As, Cd, Ba, La, Pb.
Data source location	Pattipulam to Devanampattinam, East Coast of Tamilnadu, India
Data accessibility	Data is with this article

**Value of the data**

• Data presented on the distribution, enrichment and sources of heavy metals in sediments can be useful to draw a base line data in marine environment.
• Data shown here used as a tool for anthropogenic causes in heavy metal content and to identify common pollution sources.
• Data shows that continuous monitoring and efforts of remediation might be required to improve the coastal environment near industrialized areas.

## 1 Data

**Fig. 1:**
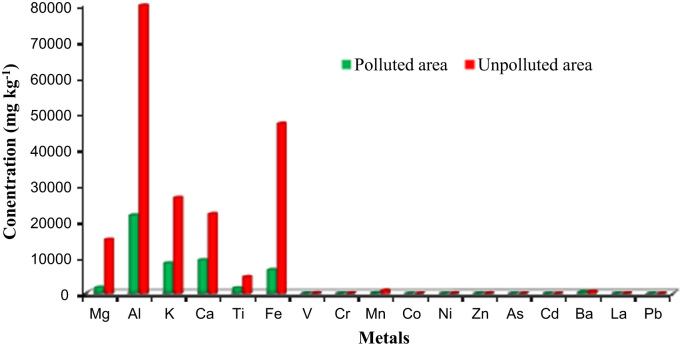
Comparison of metals concentration levels in the sediment samples in polluted and unpolluted areas.

**Fig. 2:**
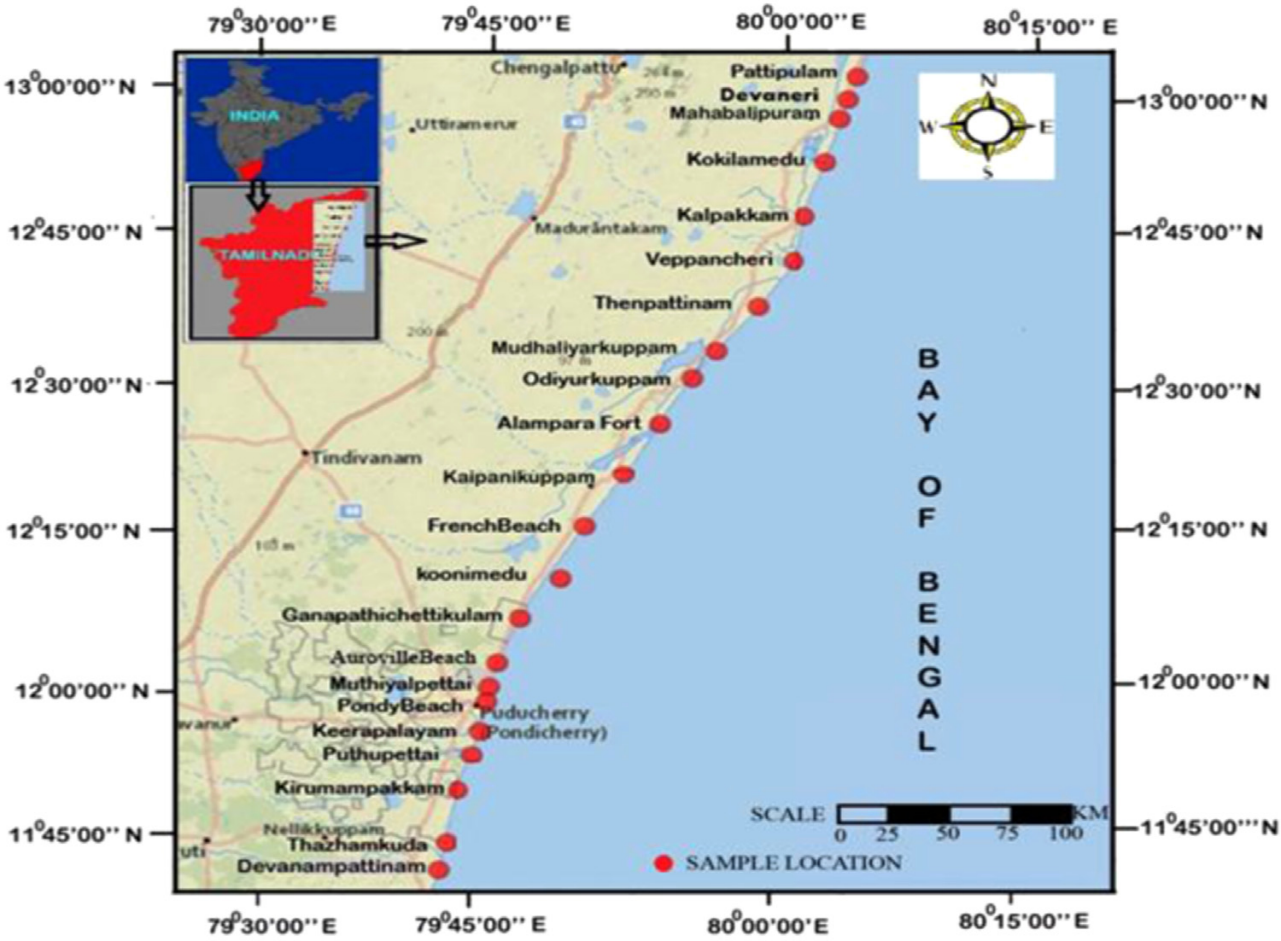
Location map.

The chemical data in sediments from Pattipulam to Devanampattinam along the East Coast of Tamilnadu is presented in Table 1. Table 2 gives the data on geographical information of the sampling points of the study area. Fig. 1 shows metals concentration levels in the sediment samples in polluted and unpolluted areas, and Fig. 2 shows the sampling points in the study area.

## 2 Experimental design, materials and methods

### 2.1 Sample collections

Sediment samples were collected along the Bay of Bengal coastline, from Pattipulam to Devanampattinam coast during pre-monsoon condition. These samples were collected in pre-monsoon season, when sediment texture and ecological conditions can be clearly observed, when erosional activities are predominant, and sediments were not transported from the river and estuary towards the beach and marine. In order to ensure minimum disturbance of the upper layer, samples were collected by a Peterson grab sampler from 10 m water depths parallel to the shoreline. The grab sampler collects 10 cm thick bottom sediment layer from the seabed along the 22 stations.

Uniform quantity (about 2 kg) of sediment samples were collected from all the sampling stations. Sampling locations were selected to collect representative samples from all along the study area. Table 2 represents the geographical latitude and longitude for all the sampling locations of the study area. Care was taken to ensure that the collected sediments were not in contact with the metallic dredge of the sampler, and the top sediment layer was scooped with an acid washed plastic spatula. Sediment samples were stored in refrigerated at −4 °C until analysis [2]. Then pebbles, leaves and other foreign particles were removed.

### 2.2 EDXRF analysis

The samples were air dried at a temperature of 110 °C until constant weight, lightly ground in an agate mortar for homogenization and sieved to pass <63 µm (metals are most often associated with small grains) [3]. All powder samples were stored in desiccators until they were analyzed. One gram of the fine ground sample and 0.5 g of the boric acid (H_3_BO_3_) were mixed. The mixture was thoroughly ground and pressed to a pellet of 25 mm diameter using a hydraulic press (20 tons) for EDXRF analysis. Table 3 list the analysis of soil standard-2709a reference sample using EDXRF.

## Conﬂict of interest

The authors declare no conflict of interest associated with this manuscript.

## Figures and Tables

**Table 1:** Heavy metal concentration (mg kg^−^^1^) in sediments from East coast of Tamilnadu, India.

**S. No**	**Location ID**	**Location**	**Mg**	**Al**	**K**	**Ca**	**Ti**	**Fe**	**V**	**Cr**	**Mn**	**Co**	**Ni**	**Zn**	**As**	**Cd**	**Ba**	**La**	**Pb**
1.	PPM	Pattipulam	500	21,722	9200	8550	1043	5486	26.9	29.9	108.8	2.1	18.0	66.0	6.2	4.9	422.9	9.9	12.9
2.	DVN	Devaneri	2600	27,900	8900	11,000	9889	21,836	162.2	61.9	386.9	7.1	20.3	58.7	7.0	3.2	362.8	123	14
3.	MAM	Mahabalipuram	900	21,800	8300	9800	2122	9138	45.8	31.6	178.3	3.3	18.7	34.1	5.8	0	435.8	10.2	6.1
4.	KKM	Kokilamedu	1500	24,000	9300	10,500	1911	7557	35.6	26.3	156.5	2.7	18.1	27.9	5.6	4.7	485.2	21.8	6.1
5.	KPM	Kalpakkam	900	22,600	9300	9500	1352	6396	30.5	28.0	120.5	2.1	18.6	36.2	5.7	3.9	434.4	20.4	10
6.	VPC	Veppancheri	2500	24,900	9000	9800	1614	7355	34.2	29.4	138.5	2.7	20.0	36.5	5.3	7.5	453.8	18.1	8.9
7.	TPM	Thenpattinam	4200	23,700	8700	10,800	1543	7423	35.6	38.6	153.5	2.8	24.0	120.3	8.4	2.6	434.6	18.7	25.8
8.	MKM	Mudaliyarkuppam	20	17,700	7200	7000	779	3945	23.8	21.7	88.9	1.4	19.7	50.1	5.5	0	302.9	7.5	7
9.	OKM	Odiyurkuppam	200	18,300	8100	8100	661	4036	24.0	21.4	81.6	1.4	16.6	22.7	4.9	2.7	421.5	0	3.8
10.	APT	Alampara fort	2400	23,200	8600	10,400	859	5513	28.0	23.6	113.2	2.1	20.6	44.2	5.5	4.6	436	2.4	7.3
11.	KPK	Kaipanikuppam	600	17,800	7400	6400	614	3532	23.2	19.2	77.2	1.2	16.3	34.9	4.7	5.2	348.9	6	2.3
12.	FBH	French beach	2600	20,200	7300	8500	1242	6283	29.5	27.8	136.5	2.3	19.3	34.9	4.7	7.1	335.4	4.8	4.5
13.	KMU	Koonimedu	2500	21,800	7900	9400	1089	5694	28.2	32.3	123.9	2.1	21.8	121.9	8.3	0.2	337.1	1.1	24.7
14.	GCM	Ganapathichettikulam	1600	19,900	7800	8100	845	4509	24.7	29.3	95.9	1.6	21.5	116.8	7.2	0	373.2	15.5	22.5
15.	ABH	Auroville beach	1500	21,700	9100	10,400	1027	5431	26.1	26.7	109.2	2.0	18.3	44.2	5.6	4.8	426.8	12.7	7.5
16.	MPT	Muthiyalpet	1600	21,500	8900	9300	942	4866	25.3	24.6	103.1	1.7	24.3	66.3	6.7	0	402.5	6.9	14
17.	PBH	Pondy beach	1300	22,600	9300	9600	772	4649	24.9	25.9	95.1	1.8	25.8	83.8	6.7	2.5	442.4	10.8	18.6
18.	KEP	Keerapalayam	1800	26,100	9500	12,500	753	5303	26.6	26.3	115.8	2.0	22.8	87.6	7	13.7	433.4	2.6	16.4
19.	PPT	Puthupettai	1800	20,000	8000	8400	945	5433	27.3	30.7	102.0	2.1	19.1	65.9	6.3	0	450.4	13.2	14.2
20.	KIP	Kirumampakkam	2500	23,100	7700	10,700	1632	8982	36.9	40.7	184.0	3.3	21.4	53.8	5.5	3.7	373.9	2.4	9.6
21.	TKA	Thazhankuda	1900	15,800	7600	5800	376	3215	22.7	19.8	61.4	1.1	17.9	91.1	6.7	2.5	393.2	3	18.7
22.	DPM	Dhevanampattinam	1200	21,500	7800	9700	1422	7604	35.0	45.6	138.3	2.9	22.1	70.3	6.3	1.5	401.6	20.2	11.7
**Average**			1665	21,719	8405	9284	1520	6554	35.3	30.1	130.4	2.4	20.2	62.2	6.2	3.4	404.9	15.1	12.1
**Minimum**			20	15,800	7200	5800	376	3215	22.7	19.2	61.4	1.1	16.3	22.7	4.7	0	302.9	0	2.3
**Maximum**			4220	27,900	9500	12,500	9889	21,836	162.2	61.9	386.9	7.1	25.8	121.9	8.4	13.7	485.2	123	25.8
**Crustal Average****[3]**			**15,000**	**80,000**	**26,600**	**22,100**	**4600**	**47,200**	**130**	**90**	**850**	**19**	**68**	**95**	**13**	**0.3**	**580**	**92**	**20**

**Table 2:** The Geographical latitude and longitude for the sampling locations of the study area.

**S. No**	**Location**	**Location ID**	**Latitude (N)**	**Longitude (E)**
1	Pattipulam	PPM	12°40׳51.27"N	80°15׳19.35"E
2	Devaneri	DVN	12°39׳19.32"N	80°14׳49.68"E
3	Mahabalipuram	MAM	12°37׳55.53"N	80°14׳13.14"E
4	Kokilamedu	KKM	12°34׳56.33"N	80°13׳22.37"E
5	Kalpakkam	KPM	12°30׳57.52"N	80°11׳50.57"E
6	Veppancheri	VPC	12°27׳58.97"N	80°11׳16.29"E
7	Thenpattinam	TPM	12°24׳42.28"N	80° 9׳48.29"E
8	Mudaliyarkuppam	MKM	12°21׳26.51"N	80° 6׳52.67"E
9	Odiyurkuppam	OKM	12°19׳35.89"N	80° 5׳44.70"E
10	Alampara fort	APT	12°16׳19.80"N	80° 3׳16.00"E
11	Kaipanikuppam	KPK	12°12׳42.65"N	80° 1׳32.40"E
12	French beach	FBH	12° 9׳2.75"N	79°59׳11.44"E
13	Koonimedu	KMU	12° 4׳59.37"N	79°55׳53.55"E
14	Ganapathichettikulam	GCM	12° 2׳45.84"N	79°56׳46.86"E
15	Auroville beach	ABH	11°59׳51.98"N	79°55׳31.39"E
16	Muthiyalpet	MPT	11°57׳43.22"N	79°52׳42.65"E
17	Pondy beach	PBH	11°56׳38.16"N	79°52׳17.45"E
18	Keerapalayam	KEP	11°54׳23.61"N	79°51׳49.37"E
19	Puthupettai	PPT	11°52׳45.44"N	79°51׳19.75"E
20	Kirumampakkam	KIP	11°50׳23.50"N	79°51׳54.44"E
21	Thazhankuda	TKA	11°46׳28.21"N	79°49׳31.03"E
22	Dhevanampattinam	DPM	11°44׳41.37"N	79°49׳23.01"E

**Table 3:** Results obtained from the analysis of soil standard-2709a reference sample using EDXRF (in mg kg^−1^).

**Element**	**Certified values**	**EDXRF values**
Mg	14,600.0	14,900.0
Al	72,100.0	68,400.0
K	20,500.0	19,100.0
Ca	19,100.0	16,500.0
Ti	3400.0	3100.0
Fe	33,600.0	33,900.0
V	110.0	98.8
Cr	130.0	112.1
Mn	529.0	568.2
Co	12.8	12.8
Ni	83.0	69.3
Zn	107.0	127.9

## References

[1] Chandramohan J., Chandrasekaran A., Senthilkumar G., Elango G., Ravisankar R. (2016). Heavy metal assessment in sediment samples collected from Pattipulam to Dhevanampattinam along the East Coast of Tamil Nadu Using EDXRF Technique. J. Heavy Metal. Toxic. Dis..

[2] Ravisankar R., Sivakumar S., Chandrasekaran A., Kanagasabapathy K.V., Prasad M.V.R., Satapathy K.K. (2015). Statistical assessment of heavy metal pollution in sediments of east coast of Tamilnadu using Energy Dispersive X-ray Fluorescence Spectroscopy (EDXRF). Appl. Radiat. Isot..

[3] Morillo J., Usero J., Gracia I. (2004). Heavy metal distribution in marine sediments from the southwest coast of Spain. Chemosphere.

