# Supplementary material for: Data on Heavy metal in coastal sediments from South East Coast of Tamilnadu, India using Energy Dispersive X-ray Fluorescence (EDXRF) Technique
Source: Data Brief. 2016 Oct 6;9:661–6. doi: 10.1016/j.dib.2016.09.047 (PMC5067090; doi:10.1016/j.dib.2016.09.047)
Supplement: Supplementary file 1 — Supplementary material [file mmc1.docx]

From

**Dr.R.Ravisankar**

Assistant Professor

Department of Physics

Government Arts College

Tiruvanamalai

E-Mail: [ravisankarphysics@gmail.com](mailto:ravisankarphysics@gmail.com)

To

**The Editor**

Data in Brief

Respected Sir

Sub: **Conﬂict of interest – Reg.**

The paper entitled “**Data on Heavy metal content in coastal sediments from South East Coast of Tamilnadu, India using EDXRF Technique”** is submitted for revision. I strongly declare that no conﬂict of interest associated with this manuscript.

Thank You

**Yours**

**(R.Raviankar)**
